# Novel Magnetic Nanostructured Beads for Cadmium(II) Removal

**DOI:** 10.3390/nano9030356

**Published:** 2019-03-04

**Authors:** Lisandra de Castro Alves, Susana Yáñez-Vilar, Yolanda Piñeiro-Redondo, José Rivas

**Affiliations:** Applied Physic Department, NANOMAG Laboratory, Research Technological Institute, Universidade de Santiago de Compostela (USC), 15782 Santiago de Compostela, Spain; lisandracristina.decastro@usc.es (L.d.C.A.); yolanda.fayoly@gmail.com (Y.P.-R.); jose.rivas@usc.es (J.R.)

**Keywords:** magnetic, nanoparticles, alginate, beads, adsorption, cadmium

## Abstract

This study presents an effective magnetic separation method for cadmium removal, based on the use of a novel nanostructured material as an adsorbent. This adsorbent involves the incorporation of magnetite nanoparticles (Fe_3_O_4_-NPs), synthesized by the reverse coprecipitation method, into sodium alginate and activated carbon to form spherical structures by crosslinking Ca^2+^ ions with the charged alginate chains, referred to as magnetic alginate activated carbon (MAAC) beads. The effect of the experimental parameters, such as pH, contacting time, adsorbent dosage, agitation type, and rotating speed were investigated and optimized for an efficient removal of Cd(II) ions at an initial concentration of 250 mg/L. The amount of adsorbed Cd(II) by MAAC beads increased at a pH of 6 with a removal efficiency over 90%. The maximum adsorption capacity reached was 70 mg/g of adsorbent at an initial Cd(II) concentration of 150 mg/L, whereas at 250 mg/L the adsorption capacity lowered until 60 mg/g. Sorption isotherms were calculated using Langmuir, Freundlich, Temkin, and Dubinin–Radushkevich equations, and were better described by the Freundlich and Temkin models. These results proved the removal efficiency and the potential use under real environmental conditions of the MAAC beads, due to their easy recovery from contaminated aqueous solutions.

## 1. Introduction

Removing toxic heavy metals from wastewater is becoming one of the greatest matters of interest due to their large toxicity in the environment and all living beings [1,2]. Heavy metals such as cadmium, mercury, nickel, lead, and chromium, present toxic effects at low concentrations since they are accumulated in living organisms, causing several disorders and diseases [3,4]. Industrial activity, such as electroplating, battery manufacturing, fertilizers, mining, and metallurgical processes among a large set, are the main causes of anthropogenic sources of wastewater polluted with considerable amounts of heavy metals [5]. Therefore, wastewater treatments, prior to their release into the environment, are a compulsory procedure that has been addressed in several ways. Common treatments, such as chemical precipitation, membrane filtration, coagulation-flocculation techniques [6,7], and ion exchange resins [3], are expensive and ineffective when low concentrations of metal ions are present in wastewaters [8,9].

Therefore, new and more effective technologies are required to reduce heavy metals to environmentally acceptable levels at a reasonable cost. In this regard, one of the most promising methods is metal biosorption, which consists in the use of natural materials from biological sources to uptake metals from solution. The main challenge consists in the development of biosorbents with high adsorption capacities and uptake velocities. Different plants, raw materials from microbiological origin, or biopolymers such as chitin, alginate, pectin, and agarose, show great affinity towards metal ions and have been tested in water originated from industrial effluents [10,11,12,13,14,15,16] with positive results. 

Among them, alginate, a natural polysaccharide extracted from brown algae and composed of β-d-mannuronic acid and α-L-guluronic acid residues, is a promising candidate since it is very cheap, non-toxic [17], and presents a large number of carboxylic and hydroxyl functional groups that provides it with high affinity and binding capacities for metal ions [18,19,20,21,22,23,24]. 

When alginate is mixed with divalent cations, such as calcium, a spontaneous cross-linking reaction occurs, and a three-dimensional hydrogel is formed as a consequence of interchain associations, allowing in this way the preparation of gel beads of different sizes by tailoring the reaction conditions. This is the main process behind the development of metal biosorbents with a large surface area [25] that can be optimized by the incorporation of other co-sorbent materials (chitosan [3], activated carbon [26], aluminum silicates [27], humic acid [28], etc.) onto the gel matrix [6,29,30,31,32].

However, the separation or extraction of metal loaded biomaterial beads from the treated aqueous media is a matter of concern that is being addressed by means of magnetic separation strategies. This technique is also used in conventional industries, like mineral separation in the steel industry or biomarker separation in biomedical detection kits. Magnetic adsorbents, also called magsorbents, are receiving considerable attention [33] since they offer the possibility not only of being quickly recovered from the medium, but also being able to be magnetically manipulated by an externally applied field, allowing, for example, magnetically forced mixing to facilitate the adsorption process. 

The magsorbents have enormous advantages with different applications, like the addition of passivating or flocculating agents with no additional pollution being produced. One of the most used strategies consists of incorporating magnetite nanoparticles (Fe_3_O_4_-NPs) into the alginate beads as the active magnetic part of the absorbent to allow for an easy separation and recovery of the beads from treated waters [34,35,36,37,38]. 

Hence, the aim of this work was to develop a nanostructured magsorbent with a nanometer-sized microstructure [39]. The material is based on the combination of superparamagnetic magnetite nanoparticles in alginate beads choosing activated carbon as the adsorbent to be used, since it is extensively applied for the removal of pollutants because of its high specific surface area and surface reactivity [27]. The efficacy of this material was studied for biosorption applications and their ability in removing Cd(II) ions from aqueous solution. Different experimental parameters, such as pH, adsorbent dose, contacting time, cadmium concentrations, agitation type, and speed were varied to study their effect in the adsorption process. 

## 2. Materials and Methods

### 2.1. Chemicals and Materials

All chemicals used were of analytical grade and without purification. Ferric chloride (FeCl_3_·6H_2_O) was obtained from Alfa Aesar (Madrid, Spain), cadmium chloride hemi(pentahydrate) 99% (CdCl_2_·2.5H_2_O) was obtained from Acros Organics (Geel, Belgium), commercial activated carbon powder (MW = 12.01 g) was obtained from PANREAC (Madrid, Spain), ferrous sulfate (FeSO_4_·7H_2_O), calcium chloride, ammonium hydroxide, and sodium alginate were purchased from Sigma (Saint Louis, MO, USA). 

### 2.2. Synthesis of Magnetic Alginate Beads

Fe_3_O_4_ NPs were synthesized by the reverse coprecipitation method [40]. In this procedure, 15 mL of 1.0 M FeCl_3_·6H_2_O and 0.5 M FeSO_4_·7H_2_O were mixed until total dissolution. This mixture was added dropwise into an ammonium hydroxide solution (20 mL, 3.5 M) at 60 °C and stirred mechanically for 30 min. The NPs were washed several times with distilled water, and re-dispersed in 150 mL of water.

Magnetic alginate beads were prepared using calcium chloride as a cross-linking agent. Firstly, 2.0 g of sodium alginate was weighted using a Mettler AT100 analytical balance, which was subsequently added to the previously prepared Fe_3_O_4_-NPs solution. Finally, 3.0 g of activated carbon powder was added. The obtained suspension was mixed for 4 h, and then added dropwise into a bath of 0.27 M calcium chloride under a continuous constant speed of 450 rpm using a New Era NE-300 syringe pump (Biogen, Madrid, Spain). Magnetic alginate activated carbon (MAAC) beads were instantly formed and left in the solution bath for hardening during 30 min. The beads were then cleaned with distilled water. Afterwards, cleaned MAAC beads were reserved in distilled water (pH = 6.5) and dried overnight at 60 °C for the adsorption experiments. 

### 2.3. Characterization of Fe_3_O_4_-NPs and MAAC Beads

The morphology of the Fe_3_O_4_-NPs and MAAC beads was characterized by transmission electron microscopy (TEM) using a JEOL JEM-1011 microscope (JEOL, Tokyo, Japan) operating at 100 kV and by scanning electron microscopy (SEM) analysis using a ZEISS FE-SEM ULTRA Plus (30 kV) microscope (Zeiss, Oberkochen, Germany). 

Structural studies were performed by X-ray diffraction (XRD) using a Philips diffractometer (Panalytical, Callo End, UK) with Cu Kα radiation (λ = 1.5406Å), with a step size of 0.02° and a counting time of 2 s per step from 10° to 80° (2*θ*). The Fourier transform infrared spectra (FT-IR) were recorded on a Varian FT-IR 670 (Varian, Palo Alto, CA, USA) spectrophotometer in the range 400–4000 cm^−1^. Thermogravimetric analyses (TGA) were performed on heating, from 50 to 840 °C, 10 mg of sample at 10 °C/min under nitrogen flow (20 mL/min) using a TGA Perkin Elmer Pyris 7 (Perkin, Waltham, MA, USA). Magnetic properties were assessed by measuring the magnetization curve using a vibrating sample magnetometer (VSM) (DMS, Massachuset, MA, USA) with an applied field between −10,000 and 10,000 Oe at room temperature.

The concentration of Cd(II) ions was measured by inductively coupled plasma optical emission spectrophotometry (ICP-OES) using an emission spectrometer Perkin Elmer Model Optima 3300 DV (Perkin, Waltham, MA, USA).

### 2.4. Batch Sorption Experiments

Several adsorption experiments were carried out, in this study, in order to investigate the optimum conditions to achieve the highest amount of Cd(II) removal. To optimize the process of removing Cd(II), experimental parameters, such as adsorbent dosage, contacting time, pH, rotating speed, and agitation type (orbital agitation, where a circular motion of the solution along the horizontal plane of the incubator IKA KS 4000i (IKA, Staufen, Germany) and magnetic stirring agitation, where the solution is moved in a vertical circular motion as result of a magnetic stirring bar). The effect of the agitation type was investigated while varying one of the following parameters: the adsorbent dosage (2.5–15 mg), the contacting time (5, 10, 15, 20, 30, 60 min), and the agitation speed (150, 250, 300, 500 rpm) using a cadmium concentration of 10 mg/L at room temperature. These optimized parameters were used to study the pH effect on cadmium adsorption by the MAAC beads. The pH range studied was varied from 3.0 to 11 and adjusted with 0.1 M HCl and 0.05 M NaCl using a pH meter Milwaukee pH51 waterproof (Aldo, Madrid, Spain). The effect of initial concentrations of Cd(II) was studied from 10 to 250 mg/L while keeping all the other optimized parameters unchanged.

The cadmium removal efficiency was calculated using the following equation:

(1) $$\%\;R=\frac{\left(C_{0}-C_{t}\right)}{C_{0}}\;\times \;100$$

where *R* is the removal efficiency; *C*_0_ (mg/L) is the initial concentration; and *C_t_* (mg/L) is the concentration at any time *t*.

The *q* value shows the metal amount adsorbed per specific adsorbent amount (mg/g). The adsorption capacity at time *t*, *q_t_* (mg/g), was obtained as follows:

(2) $$q_{t}=\frac{\left(C_{0}-C_{t}\right)V}{M}$$

where *V* (*L*) is the volume and *M* (*g*) is the dry weight of adsorbent.

The adsorption amount at equilibrium, *q_e_*, was determined using the equation:

(3) $$q_{e}=\frac{\left(C_{0}-C_{e}\right)V}{M}$$

where *C_e_* (mg/L) is the equilibrium metal concentration present in solution.

## 3. Results and Discussion

### 3.1. Characterization of the Fe_3_O_4_-NPs and MAAC Beads

The morphology of the Fe_3_O_4_-NPs was studied by TEM and the different surface views of the MAAC beads were analyzed by SEM.

**Figure 1:**
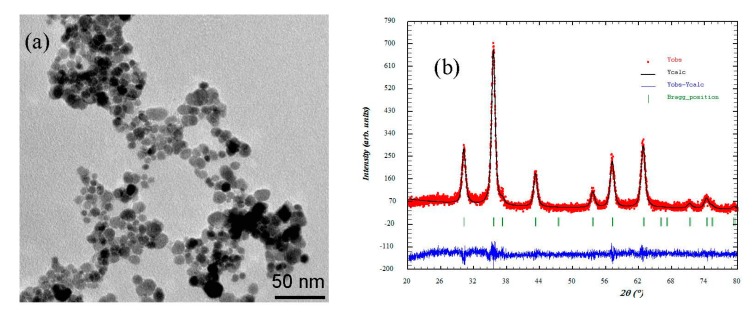
(**a**) Transmission electron microscopy (TEM) and (**b**) X-ray diffractogram adjusted by the Rietveld method of the Fe_3_O_4_- nanoparticles (NPs).

Figure 1 shows the TEM image of the Fe_3_O_4_-NPs used to synthesize the MAAC beads. The nanoparticles revealed a nanosphere morphology with an average particle size of 11 nm. Also, the average crystallite size was determined with the Scherrer equation [41]:

(4) $$L=\frac{K\lambda }{\beta cos\theta }$$

where *L* is the mean size of the ordered (crystalline) domains; *K* is a constant related to crystallite shape normally taken as 0.9; *λ* is the X-ray wavelength in nanometers; *β* is the peak width of the diffraction peak profile at half the maximum intensity (FWHM); and *θ* is the Bragg angle. 

The crystallite size calculated from the X-ray diffractogram, as shown in Figure 1b, using the Scherrer equation was 9.68 nm. The difference between TEM and X-ray diffraction can be attributed to the fact that “crystallite size” is not synonymous with “particle size”, while X-ray diffraction is sensitive to the crystallite size inside the particles. 

**Figure 2:**
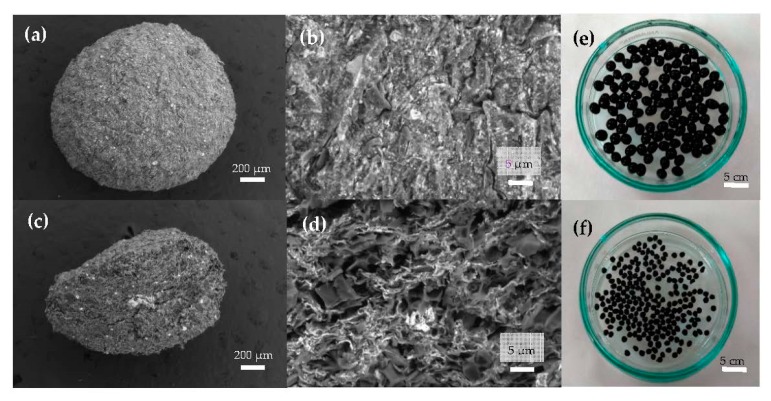
Scanning electron micrographs of the surface morphology of magnetic alginate activated carbon (MAAC) beads: (**a**) external surface, (**b**) surface interconnected pores at higher magnification, (**c**) cross-section, and (**d**) internal pore walls of MAAC. Digital images of (**e**) wet and (**f**) dry MAAC beads.

In Figure 2a, the representative SEM image of a magnetic bead can be observed, showing a nearly spherical morphology. Figure 2b reveals a bead surface structure with a very rough profile crowded with cracks that were caused by the shrinkage during the bead’s dehydration. The porous internal architecture has been analyzed by cutting the beads into two halves and performing SEM analyses of the internal flat surface, as shown in Figure 2c. In Figure 2d, at a smaller length scale, a random distribution of interconnected pores with the presence of magnetite (white spots) embedded in the pore walls of the MAAC beads can be observed. A photograph of the wet beads after the synthesis procedure has been included in Figure 2e, where their homogenous shape and size (diameter of about 2.0–3.5 mm) together with the black color can be noticed, arising from the presence of Fe_3_O_4_-NPs. In Figure 2f, the same beads after a drying procedure for storing purposes show an irregular spherical shape and a decreased diameter of approximately 1.3 mm. 

**Figure 3:**
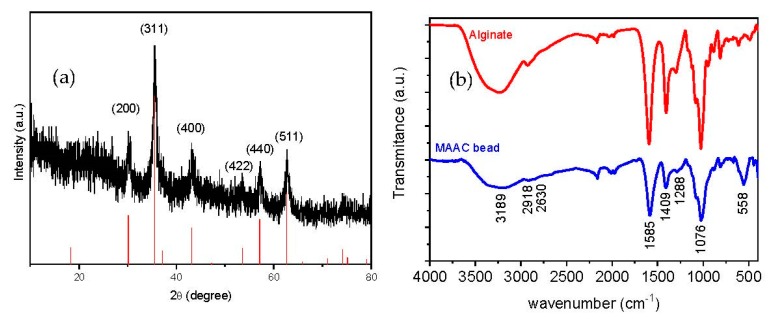
(**a**) X-ray diffractogram of the MAAC beads and standard XRD of Fe_3_O_4_ and (**b**) IR spectra of sodium alginate and MAAC beads.

The X-ray diffraction pattern of the dried beads is shown in Figure 3a, where six characteristic peaks appearing at 2*θ* = 30.1, 35.8, 43.1, 53.9, 57.2, and 63.1° can be indexed to (200), (311), (400), (422), (440), and (511) planes corresponding to the magnetite crystalline phase (JCPDS 79-0417). The relative intensity and locations of the peaks, similar to the theoretical pattern of magnetite, clearly evidence that the crystallinity nature of Fe_3_O_4_-NPs embedded in the MAAC beads structure is preserved during all the synthesis procedures.

Chemical topography of the MAAC beads is analyzed by FT-IR spectrometry as shown in Figure 3b. The broad peak at 3189 cm^−1^ corresponds to the –OH stretching vibration of alginate; the peaks at 2918 and 2630 cm^−1^ belong to symmetric and asymmetric C–H stretching, respectively. The bands which appeared at 1585 and 1409 cm^−1^ are assigned to asymmetric and symmetric stretching vibrations of carboxyl groups of alginate, respectively. The band at 1076 cm^−1^ is attributed to C–O–C groups [42]. Another important band was detected at 558 cm^−^^1^ which corresponds to the stretching vibration of Fe–O bonds in Fe_3_O_4_-NPs [43]. It can be assumed from FT-IR spectra that strong bonding has occurred between alginate and the commercial activated carbon due to the downshift observed in the alginate band from 1593 to 1585 cm^−^^1^ [44]. 

**Figure 4:**
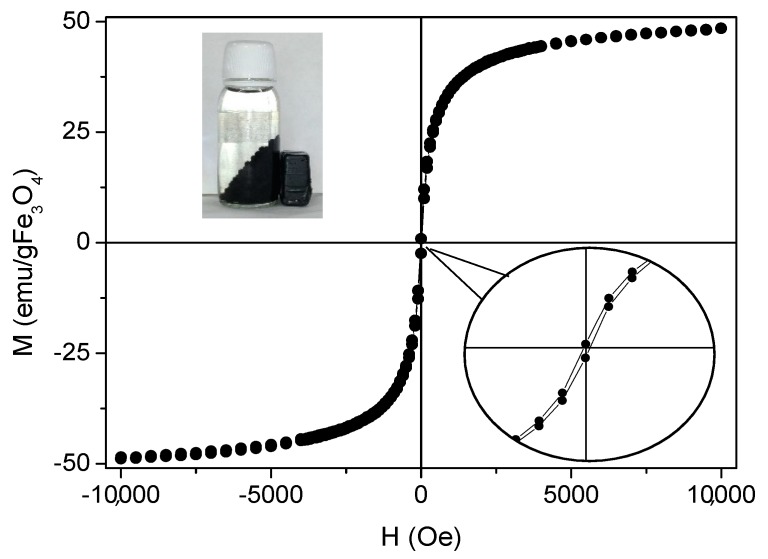
Magnetization curve of MAAC beads at 25 °C. Insets: amplification of hysteresis cycle and digital photograph when an external magnet is applied to the MAAC beads.

Magnetization of the beads was measured with a vibrating sample magnetometer working from −10 kOe to 10 kOe at room temperature. The results were normalized to the amount of magnetic mass content of the sample, which was obtained by TGA analysis. The percentage of magnetite in the sample was 22.84%. In Figure 4, the magnetization cycle of a representative MAAC sample shows negligible coercivity and absent remanence which can be ascribed to the superparamagnetic (SPM) behavior of small magnetite nanoparticles below the critical size (Diameter = 30 nm). NPs in the SPM regime do not present net magnetization in the absence of an external applied magnetic field, preventing magnetic aggregation and subsequent flocculation. In addition, they facilitate individual and homogeneous dispersion in the liquid medium.

Saturation magnetization (M_S_) of the MAAC beads shows a value of 48 emu/g Fe_3_O_4_, which is smaller than bulk magnetite (M_S-bulk_ = 92 emu/g). This is in accordance to what can be expected for small nanoparticles where magnetization becomes reduced by the presence of a dead magnetic layer. Nevertheless, the net magnetization of the MAAC beads was higher when exposed to an external magnetic field due to the contribution of the large number of Fe_3_O_4_-NPs contained in each bead. This fact ensures their strong drag to an external applied permanent magnet and facilitates an almost instantaneous separation from the aqueous medium, as shown in the inset of Figure 4, or a vigorous stirring under magnetic agitation.

### 3.2. Influence of Experimental Parameters on Cd(II) Removal by MAAC Beads

#### 3.2.1. Effect of the Contacting Time

**Figure 5:**
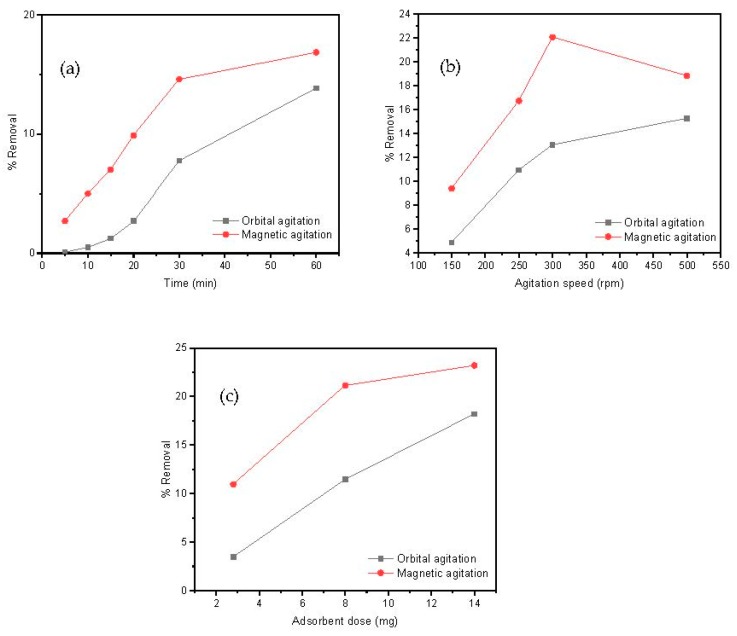
Parameters’ influence on Cd(II) adsorption: (**a**) contacting time, (**b**) agitation speed, and (**c**) adsorbent dose.

The time interval that adsorbate and adsorbent are maintained in contact is an important parameter for a designed adsorption process. For this aim, 20 mL of a Cd(II) solution (10 mg/L) and 8 mg of adsorbent, at pH = 7, were kept under orbital or magnetic agitation for distinct times to measure subsequent aliquots of metal removal at an agitation speed of 250 rpm. The effect of the contacting time on the removal efficiency of Cd(II) by the MAAC beads at different times is shown in Figure 5a. Independently to the contacting time, magnetic agitation was more efficient than orbital mixing in metal removal. After 10 min, a Cd(II) removal of approximately 5% in the magnetic agitation was observed, while approximately 0.1% Cd(II) removal was observed in the orbital case. This initial adsorption indicates a favorable motion by stirring, which allowed an easy adsorption and penetration onto the surface sites of the MAAC beads. As the time proceeded, the Cd(II) ions accumulated over the absorbent’s surface and the removal percentage constantly increased. After 1 h, approximately 18% and 12% of Cd(II) was removed in both the magnetic and orbital agitation, respectively. This contacting time of 1 h will be used as a fixed value in the subsequent experiments done in the present study. 

#### 3.2.2. Effect of the Rotating Speed and Type

The influence of the rotating speed on the adsorption process was also studied by analyzing the metal removal for magnetic and orbital agitation from 150 to 500 rpm. The results, as shown in Figure 5b, show that the removal efficiency of Cd(II) increased with the agitation speed in both orbital and magnetic agitations for 1 h. A maximum Cd(II) removal of 22% was observed in the magnetic agitation at 300 rpm, whereas throughout the adsorption process in the orbital agitation, the removal percentage of Cd(II) remained lower than 15%. These results suggest that, for the above described experimental set up conditions, the optimum agitation speed was 300 rpm using the magnetic agitation and it will be the fixed parameter.

Magnetic agitation of the MAAC beads has shown to be generally more efficient than orbital agitation, as shown in Figure 5a–c. One of the reasons for this result is the fact that the magnetic agitation is provoked by an external rotating magnetic field that affects all the magnetic beads equally in the solution. This is of crucial industrial importance, since magnetic agitation can be much more easily implemented than orbital agitation for large wastewater reservoirs. 

#### 3.2.3. Effect of the Adsorbent Dosage

In addition, the influence between adsorbent and adsorbed materials on the removal procedure was assessed by keeping constant all the experimental parameters (contacting time of 1 h, agitation speed of 300 rpm) while varying the mass of MAAC beads between 2 and 15 mg. This adsorbent dose range was selected as a criterion for successful employment of this material to industrial applications by reducing costs using minimum adsorbent dosages. In Figure 5c, a direct correlation between the rising of Cd(II) removal and the increase in amount of added adsorbent can be observed, which is a reasonable result taking into account that an increment of total surface sites available for metal loading allows for a larger loading capacity. The loading saturation capacity was not achieved for the used adsorbent dosages. A maximum Cd(II) removal at 14 mg of approximately 24% and 16% was observed at the magnetic and orbital agitation, respectively. Therefore, according to these results, the adsorbent dose selected was 14 mg using the magnetic agitation. 

#### 3.2.4. Effect of pH on Cd(II) Adsorption

The solution pH is one of the most important parameters able to govern metal adsorption, since it acts on the protonation of the binding sites of the adsorbent and consequently strongly affects the adsorption capacity [45]. The influence of pH on Cd(II) uptake capacity by MAAC beads was studied at pH values ranging from 3.0 to 11 using the optimized parameters of the previous studies, which were magnetic agitation at 300 rpm, using 14 mg of dried MAAC beads for 1 h.

**Figure 6:**
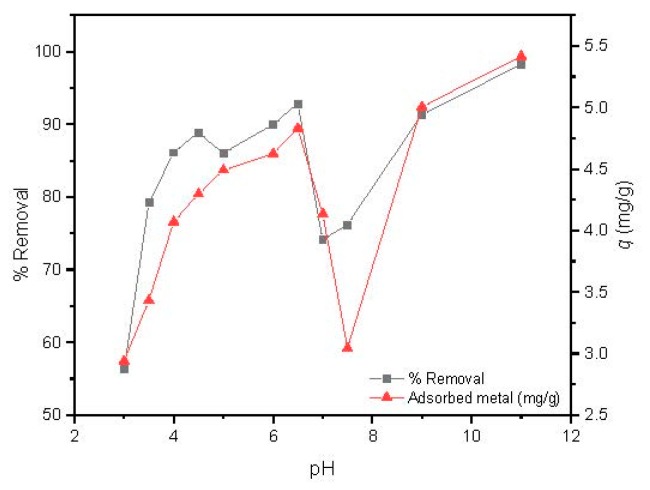
Influence of pH on Cd(II) adsorption by MAAC beads.

As observed in Figure 6, the Cd(II) removal percentage increases with the pH values from 3 to 6.5, followed by a steep decrease with a minimum removal and adsorbed metal around pH = 7.5, and a further increase up to a pH value of 11. It has to be highlighted that for pH values over 6.5, a large precipitation of cadmium was observed due to the formation of several hydroxyl low soluble species, such as Cd(OH)_2_ and Cd(OH)_3_ [45].

The adsorption capacity of Cd(II) by MAAC beads at a pH value of 3–4 attains removal efficiency up to 78% which can be attributed to an insufficient number of deprotonated sites, arising from the competition of available binding sites between H^+^ and Cd(II) ions [45]. By increasing the pH range from 5 to 6.5, a continuous increase in the adsorption capacity was observed up to almost 95% of removal, with 4.3 to 4.8 mg/g of adsorbed Cd(II) ions per adsorbent mass. At this pH range, the adsorbent surface became negatively charged due to higher deprotonation, which enhanced the adsorption of Cd(II) ions through electrostatic attraction forces [46]. This result is of large industrial interest, since slight modifications of pH can be used as a strategy for obtaining high removal efficiencies. For the next experiments, we fixed the pH in the Cd(II) removal to 6, which provided a removal efficiency over 90%.

#### 3.2.5. Effect of Cd(II) Initial Concentration

**Figure 7:**
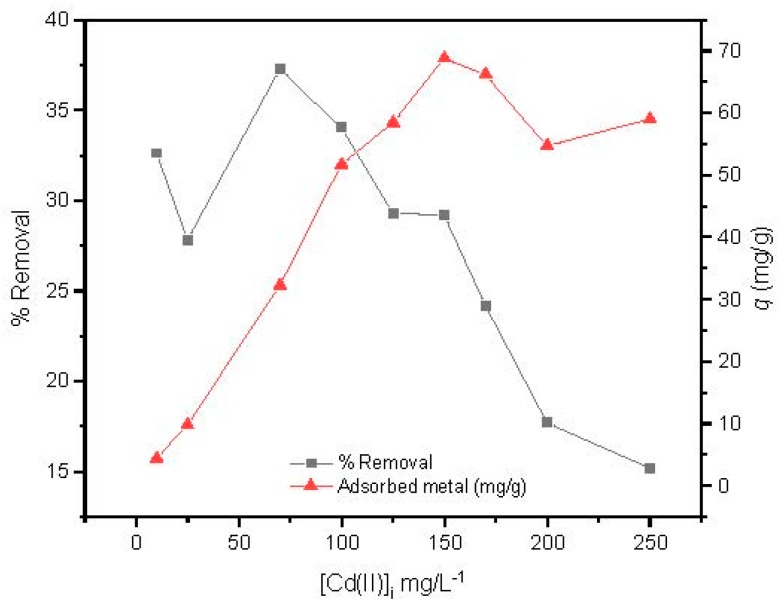
Effect of Cd(II) initial concentration.

The effect of the Cd(II) initial concentration was studied from 10 to 250 mg/L at optimized conditions studied previously (magnetic agitation at 300 rpm, using 14 mg of MAAC beads during 1 h at pH = 6). In Figure 7, it is shown that the adsorption capacity increased with the increase in initial concentration, reaching its maximum adsorption at 150 mg/L. After that, a slight and constant decrease of the adsorption capacity was observed, revealing that a plateau had been reached.

Conversely, the removal efficiency of Cd(II) by the MAAC beads decreased with increasing initial cadmium concentration in solution. This may be due to the saturation adsorption sites of the MAAC beads throughout the adsorption process, leading to a reduction uptake with increasing Cd(II) initial concentration. 

#### 3.2.6. Sorption Equilibrium Isotherm

In general, the adsorption isotherm describes the mobility of a substance from an aqueous media to a solid-phase at constant conditions of temperature and pH. The equilibrium is only established when a dynamic balance is reached between the adsorbed substance onto the adsorbent surface and the remaining one in solution. This phenomenon can be described by several isotherm models, depending on the specific details of the adsorption process, the adsorbent properties, and the adsorbed species, like Langmuir, Freundlich, Temkin, or Dubinin–Radushkevich isotherms.

The Langmuir isotherm assumes monolayer and homogeneous adsorption onto a surface containing a finite number of adsorption sites that possess equal affinity for the adsorbate [47,48]. Once a site is filled, no further adsorption can occur at that site, indicating that the surface can reach a saturation point where the maximum surface adsorption will be achieved [49]. The isotherm is expressed by the following non-linear equation [47]:

(5) $$q_{e}=\frac{q_{m}K_{L}C_{e}}{1+K_{L}C_{e}}.$$

The linear form of the Langmuir isotherm is given by [47]:

(6) $$\frac{C_{e}}{q_{e}}=\frac{1}{K_{L}q_{m}}+\frac{C_{e}}{q_{m}}$$

where *q_e_* (mg/g) is the amount adsorbed; *C_e_* (mg/L) is the adsorbate concentration in solution, both at equilibrium; *K_L_* (L/mg) is the Langmuir adsorption constant; and *q_m_* (mg/g) is the maximum sorption capacity for monolayer formation on the adsorbent.

Hereby, a fundamental characteristic of the Langmuir isotherm regarding a dimensionless constant, known as separation factor *R_L_*, defined by Weber and Chakraborti in 1974, can be represented as [47]:

(7) $$R_{L}=\frac{1}{1+K_{L}C_{0}}$$

where *C*_0_ (mg/mL) is the adsorbate initial concentration.

The Freundlich isotherm assumes that the adsorbent has an energetically heterogeneous surface and has a non-uniform distribution for adsorption. The isotherm is represented by the non-linear equation [50]:

(8) $$q_{e}=\;K_{F}C_{e}^{1/n}.$$

The linear form of the Freundlich isotherm is given by [50]:

(9) $$\log q_{e}=\log K_{F}+\frac{1}{n}logC_{e}$$

where 𝐾_F_ and 𝑛 are empirical constants, being indicative of the adsorption extent and the *f* nonlinearity degree between solution and concentration, respectively. 

The Temkin isotherm model is assumed by the ratio between the adsorption heat and adsorbent surface coverage [47]. The adsorption heat of all molecules present in the layer decreases linearly with the coverage because of adsorbent–adsorbate interactions. The model accepts a uniform distribution of binding energies as well, up to maximum binding energy [51]. The Temkin isotherm is represented by the following non-linear equation [51]:

(10) $$q_{e}=B\ln(K_{T}C_{e})$$

where B (J/mol) is the constant related to the adsorption heat and *K_T_* (L/g) is the Temkin isotherm equilibrium binding constant. 

However, the rearranged linear form of the Temkin isotherm equation is commonly used, represented by [51]:

(11) $$q_{e}=\frac{RT}{b_{T}}\ln K_{T}+\frac{RT}{b_{T}}ln\;C_{e}$$

where *R* (8.314 J/mol·K) is the universal gas constant; *T* (*K*) is the absolute temperature; and *b_T_* is the Temkin isotherm constant.

The Dubinin–Radushkevich (D–R) isotherm model is usually applied to describe the characteristic porosity of the adsorbent and adsorption energy. The non-linear form is expressed as [52]:

(12) $$q_{e}=\;q_{m}\exp\left(-\beta \varepsilon^{2}\right)$$

where *q_m_* (mg/g) is the adsorption capacity; *β* is the activity coefficient pertinent to mean adsorption energy; and *ε* is the Polanyi potential, which can be calculated through Equation (13) [52]:

(13) $$\varepsilon =\mathrm{RT}\ln(1+\frac{1}{C_{e}}).$$

The linear form of this model is described as [52]:

(14) $$\ln q_{e}=\ln q_{m}-\beta \varepsilon^{2}.$$

However, it also specifies the nature of the adsorption process through physisorption or chemisorption of metal ions, with its mean free energy, *E*, per molecule of adsorbate can be calculated by the relationship (kJ/mol) [52]:

(15) $$E=\frac{1}{\sqrt{2\beta }}.$$

The adsorption equilibrium was studied using the following optimized parameters: magnetic agitation with a rotating speed of 300 rpm, 14 mg of MAAC beads, contacting time of 1 h, and pH = 6. 

**Figure 8:**
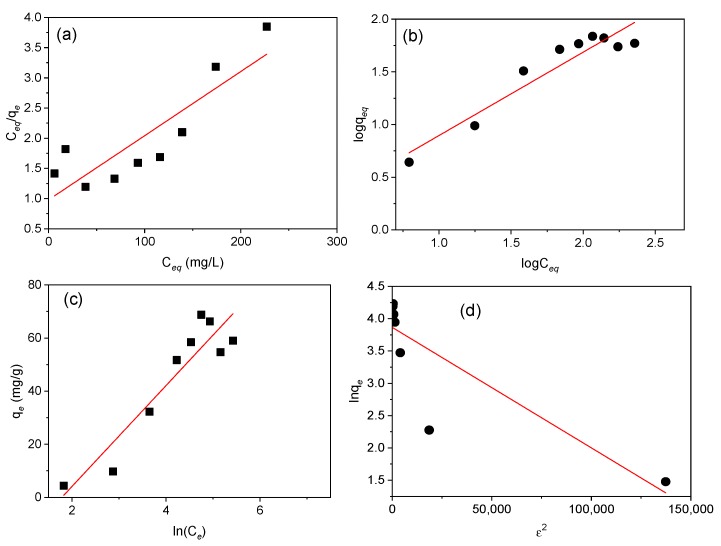
Langmuir, Freundlich, Temkin, and Dubinin–Radushkevich isotherm plots. (**a**) Langmuir isotherm showing the adsorption variation (*C_e_*/*q_e_*) against the equilibrium concentration (Ce), (**b**) Freundlich isotherm representing variation of log*q_e_* against log*C_e_*, (**c**) Temkin isotherm plot of *q_e_* (mg/g) against ln(*C_e_*), and (**d**) Dubinin–Radushkevich isotherm representation of ln*q_e_* against *ε*^2^ for adsorption of Cd(II) onto MAAC beads for 1 h.

The Langmuir, Freundlich, Temkin, and D-R parameters of Cd(II) adsorption onto MAAC beads were obtained and listed in Table 1 and represented in Figure 8.

The comparison of R^2^_,_ represented in Table 1, supports that the Temkin and Freundlich models described better the equilibrium data compared to the D–R and Langmuir models. Based on the linear plot obtained from the Temkin model, the *B* index and *K_T_* constant were 18.9 J/mol and 0.169 L/g, which is an indication of the adsorption heat and physical adsorption process. According to the Freundlich model, the value of 1/*n* = 0.794 (*n* = 1.26), indicates a physical and favorable adsorption of Cd(II) onto the MAAC beads with an adsorption capacity of 1.27 mg/g [53,54]. The Freundlich isotherm, despite being able to provide us with information on surface heterogeneity and exponential distribution of the active sites and energies, cannot prevent any saturation of the adsorbent surface by the adsorbate. Therefore, based on the D–R isotherm, the obtained adsorption energy, *E*, was 1.581 KJ/mol, suggesting that the uptake of Cd(II) by MAAC beads was by physisorption with a maximum monolayer adsorption capacity of 47.68 mg/g. 

Parameter *R_L_*, with a value of 0.333, indicates a favorable equilibrium, but the very low value of R^2^ of 0.753 reveals that the sorption cannot be described by the Langmuir isotherm model. Moreover, the *q_m_* calculated was 94.34 mg/g, revealing a high Cd(II) adsorption.

Metal ion adsorption by an adsorbent is influenced by several factors, such as the metal ion size, the number of active groups and pore size present on the surface of the adsorbent, and the experimental conditions in the adsorption process (pH, concentration, contacting time, agitation type, and rotating speed).

In our case, Cd(II) adsorption is better described by the Freundlich and Temkin isotherms, which is in accordance with the multi-layer structure adsorption head, and heterogenous surface with different affinity sites on the MAAC beads. These results suggest that the adsorption follows a physical process, although to further understand the underlying mechanism, more studies are needed. 

## 4. Conclusions

The present work presents a cost-effective method for Cd(II) removal from aqueous medium by developing a novel magnetic hybrid nanostructured material through the encapsulation of Fe_3_O_4_-NPs with activated carbon in alginate-based beads. The magnetic bead structure observed by SEM analysis showed a high number of inter-connected pores and a large specific surface area, which is the reason for fast and efficient adsorption of Cd(II) ions from solution. The prepared beads showed superparamagnetic behavior, with well distributed Fe_3_O_4_-NPs and activated carbon on the beads’ structure. The effects of contacting time, initial Cd(II) concentration, pH, adsorbent dose, agitation type, and rotating speed on the adsorption process were discussed. All procedures showed a higher removal efficiency of Cd(II) when beads were under magnetic agitation. At pH 6, the adsorption isotherm that better described the study equilibrium was the Freundlich and Temkin isotherms, with R^2^ > 0.90 and R^2^ = 0.89, respectively.

Magnetic hybrid beads are good candidates for efficient magnetic removal of Cd(II) and other heavy metals present in wastewaters, being useful for industrial applications since they combine a set of advantageous features: biocompatibility, cost-effective synthetic production, easy handling, and fast separation from any aqueous solution under an external magnetic force. Future work is intended to use this material in real environmental conditions and at an industrial scale.

## Figures and Tables

**Table 1:** Langmuir, Freundlich, Temkin, and Dubinin–Radushkevich constants calculated from linear isotherms at pH 6.

Isotherm Model	Estimated Isotherm Parameters			
	*R_L_*	*q_m_* (mg/g)	*K_L_* (L/mg)	R^2^
**Langmuir**	0.333	94.34	0.01	0.753
	***N***	**1/*n***	***K_F_* (mg^1−(1/n)^L^1/n^g^−1^)**	**R^2^**
**Freundlich**	1.26	0.794	1.27	0.90
	***b_T_***	***B* (J/mol)**	***K_T_* (L/g)**	**R^2^**
**Temkin**	130.52	18.9	0.169	0.87
	***E* (kJ/mol)**	***q_m_* (mg/g)**	**Β**	**R^2^**
**Dubinin-Radushkevich**	1.581	47.68	2.00 × 10^−5^	0.733
